# Bond strength durability of three bioactive restorative materials to silver diamine fluoride treated artificially demineralized dentine

**DOI:** 10.1186/s12903-025-06552-8

**Published:** 2025-07-17

**Authors:** Mostafa A. Abdelshafi, Hanan A.N. Soliman, Dina Abdelaziz

**Affiliations:** 1 Dental Biomaterials Dep, Faculty of Dentistry, Mansoura University, Mansoura, Egypt; 2 Conservative Dentistry Dep, Faculty of Dentistry, Kafrelsheikh University, Kafrelsheikh, Egypt

**Keywords:** Alkasite, Glass hybrid, Glass ionomer, Resin-modified glass ionomer, SDF, Microtensile

## Abstract

**Background:**

This study aimed to assess the impact of 38% silver diamine fluoride (SDF) treatment regarding microtensile bond strength of three bioactive restorative materials to artificially induced caries-affected dentine after thermocycling; Alkasite restorative material, Glass hybrid restorative (GHR) and Resin modified glass ionomer (RMGI).

**Methods:**

A total of 60 artificially demineralized human mid-coronal dentine specimens were randomly allocated into two main groups (*n* = 30 each): a control group and an SDF-treated group (38% SDF). Each group was additionally split into three subgroups (*n* = 10) regarding the type of restorative material (Alkasite; Cention N, GHR; EQUIA Forte fil, RMGIC; Fuji II LC). 10,000 cycles of thermal cycling were conducted alternating between 5 ºC − 55 ºC with a dwell-duration of 15 s. µTBS was assessed with the aid of a universal testing machine (Instron Co., Canton, MA-USA) occupied with a 50 kg load cell, operating at 0.5 mm/min cross-head speed till failure. µTBS results, measured in MPa, were collected and statistically analyzed with two- and one-way ANOVA succeeded by Tukey’s post hoc test and student’s t-test (α = 0.05).

**Results:**

There was a significant reduction in µTBS after SDF application in all tested groups (*p* ≤ 0.05). Cention N recorded the highest bond strength values in both control and SDF-treated conditions, while EQUIA Forte Fil had the lowest.

**Conclusions:**

Based on the limitations of this laboratory study, the application of 38% SDF directly preceding restoration with Alkasite, GHR or RMGI resulted in a significant deterioration in bond strength to artificially induced caries-affected dentine.

## Background

Dental caries is frequently described as a disease influenced by multiple factors that result in localized demineralization of the inorganic portion of hard dental tissues [1]. Academics and clinicians are progressively adopting advanced technologies besides medicines to achieve the thought of minimal invasive restorative technique. Silver diamine fluoride (SDF) is considered a noteworthy revolution, which was first marketed in the 1970s depending on its competence to seizure dental caries [2]. The 38% concentration of SDF comprises roughly 253,870 ppm silver, 44,880 ppm fluoride, with amine serving as a solvent. In terms of efficacy on caries detention and protection, SDF exhibited superior performance over fluoride varnish unaided [3]. Distinct from other fluoride-containing products, SDF encompasses silver and fluoride together. Fluorohydroxyapatite, which is the result of fluoride ion interaction with calcium and phosphorus, has lower solubility compared with hydroxyapatite [4]. Moreover, silver was recognized to possess an antimicrobial effect [5]. SDF increases the pH of the biofilm, mitigating demineralization of dentine, and effectively combat cariogenic bacteria through its antimicrobial properties, primarily accredited to silver nitrate [6, 7]. Variety of purposes of SDF usage have been presented including caries prevention [6], arresting caries [8, 9], cavity cleanser [10], tooth desensitizer [11], root canal irrigant [12], and recently presented as caries detector attributable to the blackening consequence on caries-affected dentine [13].

Cavitated carious lesions are clinically stratified into two distinct layers based on dentine texture and treatment needs: an outer zone of soft dentine, which is heavily demineralized, structurally unsound, and easily removed with minimal pressure, and an inner zone of leathery or firm dentine, which is partially demineralized, retains structural integrity, and resists hand excavation. According to current consensus recommendations, selective removal of carious tissue aims to preserve this deeper layer of remineralizable dentine “typically leathery or firm dentine” while ensuring complete removal of the outer soft dentine, thereby promoting pulpal health, sealing ability, and long-term tooth preservation [14, 15]. As such, in restorative procedures, demineralized but structurally intact dentine that can be remineralized should be preserved and often serve as the bonding substrate as an alternative of normal dentine. Nevertheless, treating carious dentine with SDF induces discoloration, resulting in blackened dentine patches. This discoloration might pose aesthetic distress. To address this esthetic concern, particularly in anterior teeth, potassium iodide (KI) is often applied immediately after SDF. The potassium iodide crystals undergo a reaction with the free silver ions in SDF, resulting in the formation of a creamy white precipitate of silver iodide (AgI). This precipitate aids in lessening the discoloration that SDF causes by preventing the formation of darker silver compounds [16, 17]. Additionally, it was stated that employing bioactive restorative materials, such as glass ionomer cement (GIC), in caries lesions treated with SDF while practicing atraumatic restorative treatment (ART) method could enhance the final aesthetic outcome [18].

Bioactive materials are uniquely own the capability of leaching particular ions at the bonding contact area, promoting therapeutic effects and probable biomineralization [19]. With regards to this, GICs are classified as ‘‘smart’’ or ‘‘bioactive’’ materials owing to their capability to leach therapeutic levels of bioactive ions including calcium, phosphate, sodium, strontium, silicate and fluoride into the nearby environment [20]. This ion release is prompted under acidic situations encouraging adhesive bonding [19]. Calcium and phosphate ions are crucial in maintaining the mineral phase (hydroxyapatite) equilibrium of hard dental tissues. Furthermore, under slightly acidic circumstances, they could prompt tooth remineralization [20]. Chemically activated GICs, often named conventional GICs, are characteristically composed of ion-dissolving glasses built on strontium or calcium aluminofluorosilicate besides weak water-soluble homopolymeric acid of polyacrylic acid (PAA), otherwise copolymer of acrylic acid, itaconic/maleic acid [21]. Despite their versatility, conventional GICs have limited mechanical properties, which limits their use in some clinical situations [22]. To address this limitation, resin-modified glass ionomer cement (RMGIC) was introduced to enhance both mechanical and physical characteristics of GIC. RMGIC not only retains the indispensable components of conventional GIC, but integrates a monomer, usually 2-hydroxyethyl methacrylate (HEMA), besides a photoinitiator as well. Hence, they exhibit dual setting reactions: characteristic acid-base reaction of glass ionomers and light-curing reaction of resin monomers [23].

Conventional GICs can be categorized into high-viscosity and low-viscosity GICs based on the powder/liquid ratio besides the quantity of Ca^+ 2^ and Al^+ 3^ ions [24]. High-viscosity GICs are materials with glasses that have been enhanced by the incorporation of strontium, bioactive glasses, or even zirconium. These enhancements give rise to quicker working and hardening times, and a noteworthy enhancement in their physical-chemical and mechanical properties, including; biocompatibility, excellent bonding to dental structures, minimum solubility, low shrinkage and the remineralization effect by the way of persistent fluoride release [25]. Lately, a glass hybrid restorative system (Equia Forte Fil) was introduced as an enhanced high viscosity GIC. This restorative system is a highly viscous bulk-fill GI enhanced with ultrafine, reactive glass particles establishing a glass hybrid (GH) restorative material with superior mechanical and physical properties [26, 27]. Furthermore, the properties of this hybrid material is further improved by applying a nano-protective coating [27]. Recently, a new dual-cured resin-based composite known as alkasite comprising alkaline fillers, has been presented. Three inorganic glasses are included in it: a basic calcium fluorosilicate glass named an “Alkasite” filler; an ionomer glass founded on a calcium barium aluminum-fluorosilicate and a traditional inert barium alumino-silicate glass [28]. Additionally, it contains ytterbium fluoride. Dimethacrylate monomers represent the liquid phase which necessitates hand mixing with filler phase. To enable bulk placement, this material can be cured through either chemical or light activated polymerization [29]. This dual-cure restorative material can balance the acidity caused by acidogenic cariogenic bacteria because of its alkaline fillers embedded within a resin methacrylate matrix and its ability to release hydroxyl ions [30].

Several studies have demonstrated that the use of SDF does not adversely affect bonding strength of restorative materials, provided that a water rinse is performed afterward. In contrast, omitting this rinsing step significantly reduces bond strength [31–34]. Additionally, the application of SDF has been reported to have a neutral effect on the adhesion of materials such as resin composites, glass ionomer cements and RMGIC, neither enhancing nor diminishing their bonding performance [32–37]. However, other studies indicated that immediate bonding to SDF-treated dentine may compromise adhesion [10, 38, 39], prompting the investigation of delayed bonding protocols [40, 41], where bonding is postponed for several days to allow chemical stabilization of the surface. In addition, mechanical surface treatments including air abrasion, light polishing, or gentle removal of the superficial SDF-treated layer have been employed to eliminate residual silver deposits and improve surface energy and micromechanical retention [40]. Currently, evidence remains limited regarding the long-term adhesion properties of restorative materials to demineralized dentine surfaces previously treated with SDF. Thus, the purpose of this study is to assess the impact of silver diamine fluoride (SDF) treatment on the microtensile bond strength of three bioactive restorative materials to artificially demineralized dentine after thermocycling; Alkasite Restorative Material (Cention N), Glass Hybrid Restorative (EQUIA Fort Fil) and Resin modified glass ionomer (Fuji II LC). The null hypothesis tested was that neither the application of silver diamine fluoride nor the type of bioactive restorative material (Cention N, EQUIA Forte Fil, or Fuji II LC) has a significant effect on the microtensile bond strength to artificially demineralized dentine.

## Methods

### Micro-tensile bond strength (µTBS) testing

#### Specimens’ Preparation and the induction of demineralized dentine (DD)

Following approval from the Ethics Committee of the Faculty of Dentistry, Kafr Elsheikh University, Egypt (Approval No. KFSIRB200-464), Freshly extracted sound human mandibular first molars, extracted for periodontal reasons, were collected from patients aged 35–60 years at the [Department of Oral and Maxillofacial Surgery, Faculty of Dentistry, Kafr Elsheikh University]. Teeth exhibiting caries, visible cracks, fractures, restorations, or developmental anomalies were excluded from the study. After cleaning to remove debris, the selected teeth were stored in 0.1% thymol solution for 30 days, then maintained in distilled water at 4 °C until use. A total of 60 teeth that met the inclusion criteria were ultimately included in the study. This sample size was determined to be adequate based on a power analysis conducted using G*Power (Düsseldorf, Germany), with an alpha level of 0.01, a power of 95%, and an estimated effect size of 0.25. To expose the bottomward dentin, a diamond microsaw (Isomet 4000: Buehler Ltd., IL-USA) coupled with a water-cooling system was utilized to cut the occlusal enamel at a right angle to teeth’s long axis. Dentine was wet ground using 340- then 600-grit silicon carbide papers till obtaining an even flat dentine surface. Each tooth’s root was dissected approximately 2 mm directly beyond the cementoenamel junction, with the dissection parallel with occlusal surface, with the same saw machine. The whole crown surfaces of each tooth were topped with two coats of a varnish resistant to acids (Revlon, Paris), excluding dentine occlusal surface. Demineralization of revealed occlusal dentine was conducted through pH-cycling approach utilizing alternating demineralizing and re-mineralizing solutions. Each specimen was alternately cycled in 10 mL of the two solutions (8 h in demineralizing solution then 16 h in re-mineralizing solution). This process was accomplished for 2 weeks at room temperature with no agitation [42].

The specimens were then randomly sorted into two main groups (*n* = 30):

- Group I: 30 specimens were restored immediately without SDF Application (control group).
- Group II: 30 specimens were treated with SDF then restored with the respective restorative material (SDF-treated group).

Each group was further subclassified into 3 subgroups, (*n* = 10) regarding the type of restorative material used:

- Subgroup 1: Alkasite Restorative Material (Cention N).
- Subgroup 2: Glass Hybrid Restorative (GHR) (EQUIA Forte Fil).
- Subgroup 3: Resin Modified Glass Ionomer (RMGI) Cement (Fuji II LC).

Details of the materials used are listed in Table 1.

**Table 1:** Materials used in the current study

Materials	Type	Composition	Manufacturer	Batch/Lot No
Advantage Arrest	Silver Diamine Fluoride (SDF) Solution	(AgNH_2_F) solution comprises: 25% Ag, 62% H_2_O, 5% F, 8% amine PH = 10	Elevate Oral Care, FL-USA	20,223
GC Cavity Conditioner	Cavity Cleaning Agent	Polyacrylic acid (20%), H_2_O, aluminum chloride hydrate.	GC; Tokyo-Japan	1,102,171
Fuji II LC	Resin Modified Glass Ionomer (RMGI) Cement	Powder: 100% fluoroaluminosilicate Liquid: 35% HEMA, 25% distilled H_2_O, 24% polyacrylic acid, 6% tartaric acid besides 0.10% camphorquinone.		2,401,251
EQUIA Forte fil	Glass Hybrid, High Viscosity Restorative System	Powder: 95% strontium fluoro-aluminosilicate glass (containing extremely reactive small particles) and 5% polyacrylic acid Liquid: 40% aqueous polyacrylic acid		240,219 A
EQUIA Forte Coat	Nanofilled Resin	Methylmethacrylate, urethane methacrylate, phosphoric ester monomer, colloidal silica, camphorquinone		2,211,221
Cention N	Alkasite Restorative Material	UDMA, Aromatic aliphatic-UDMA, DCP, PEG-400 DMA Barium aluminosilicate glass, Calcium barium aluminum fluorosilicate glass, Calciumfluoro silicate glass, Ytterbium trifluoride, Isofiller.		Z03CKR
Demineralizing Solution	-	(pH 4.4) 2.2 mM CaCl_2_, 2.2 mM NaH_2_PO_4_, 0.05 mM Acetic Acid	Manufactured in pharmaceutical laboratory, Faculty of Pharmacy Mansoura University-Egypt	
Remineralizing Solution	-	(pH of 7.2) 1.5 mM CaCl_2_, 0.9 mM NaH_2_PO_4_, 0.15 M KCl		

*HEMA*: 2-hydroxyethylmethacrylate; *UDMA*: Urethane-Dimethacrylate; *DCP*: Tricyclodecan-Dimethanol- Dimethacrylate; *PEG-400 DMA*: Polyethylene-Glycol 400-Dimethacrylate

### SDF surface pre-treatment procedure

In SDF group, one or two drops of SDF solution were added into a mixing pot and applied directly to the occlusal surface of the specimens using a micro brush for 10 s with agitation. SDF was allowed to penetrate and engage with dentine for 1 min, subsequently excess SDF was taken off using a cotton pellet, then specimens were washed with water for 30 s and air-dried for 5 s preceding the restorative procedures. This surface pre-treatment protocol was performed in accordance with both manufacturer’s instructions and previously published protocols [43–45].

### Restorative procedure

An individual T-band matrix was used and tightened around each specimen to create a mold cavity with 4–5 mm height. For Cention N specimens, the material was mixed following manufacturer instructions and inserted as one increment followed by 10 s light curing with a LED curing light (Elipar S10; 3 M ESPE Co., Seefeld, Germany) with irradiance of 1200 mW/cm^2^. For Fuji II LC and EQUIA Fort fil specimens, dentin conditioning procedure (20% polyacrylic acid (PAA)) was applied for 10 s and the surface was washed meticulously with water for 15 s followed by gentle dryness. Fuji II LC and EQUIA Fort fil capsules were mixed for 10 s at 4000 rpm by an amalgamator. Fuji II LC was applied in 2 mm increments followed by light curing for 10 s till reaching a 4–5 height. In contrast, EQUIA Fort fil was applied in a single 4–5 mm increment. Fuji II LC and EQUIA Fort fil specimens were shielded with a protecting varnish (EQUIA coat, GC, Tokyo, Japan). All specimens were preserved in distilled water at 37 °C for 24 h. All restorative procedures were performed by a single operator who had undergone training to ensure competence and procedural consistency.

### Micro-tensile bond strength (µTBS) test

Specimens were initially subjected to thermal cycling conducted within a thermocycling water tub (Thermocycling, Robota, Alexandria-Egypt) occupied with distilled water for 10,000 cycles alternating between 5^◦^C − 55 ^◦^C. Dwell duration time was15 s and the transference time was 5 s [42]. Each specimen was sliced at a right angle to the bonding interface so as to attain beams with an approximate cross-sectional area of 1.0 mm^2^ by the means of a diamond microsaw (Isomet 4000; Buehler Ltd., IL-USA) coupled with a water-cooling system. A digital caliper (Mitutoyo, Tokyo-Japan) was used to accurately determine the cross-sectional area of the bond interface to each beam. From each specimen, beams from specimen peripheries were tossed out and five central beams were randomly chosen for testing. The beams underwent µTBS testing with the aid of a universal testing machine (Instron Co., Canton, MA-USA) equipped with a 50 kg load cell, operating at a 0.5 mm/min cross-head speed till failure. µTBS was reported in MPa. A stereomicroscope (Olympus SZ1145, Olympus Optical Co. LTD-Japan) set to 40 × was used to examine the fractured surfaces after µTBS through visual examination to categorize the failure mode which happened during µTBS testing. Failures in each group were categorized into the upcoming categories: “restorative material cohesive failure”, “dentine cohesive failure”, “dentine-restorative material interface adhesive failure”, or “mixed failure [42].

### Evaluation of dentine/restorative materials interface

Specimens for dentine/restorative material interface assessment were prepared using the same methodology as µTBS. Subsequent to 10,000 cycles of thermocycling, bonded specimens were split at a right angle to the dentine/restorative material interface utilizing a diamond microsaw (Isomet 4000; Buehler Ltd., IL-USA) coupled with a water-cooling system. The segmented specimens were polished using a sequence of silicone carbide papers (600, 800,1200, 2400, and 4000-grits, Microcut; Buehler) for 10 s each under low pressure, then sputter coated with gold. The bonded-dentine interfaces were investigated using a scanning electron microscope with an accelerating voltage of 15 kV. (JEOLJSM-5500LV SEM, JEOL Ltd, Japan) together with an energy-dispersive X- ray spectrometer (EDX). Three photomicrographs of distinctive surface areas were gained at 1000× magnification and a semiquantitative chemical microanalysis was accomplished by means of EDX- SEM [46].

### Statistical analysis

Prior to conducting the tests, Shapiro Wilk test was used to verify the normality of distribution. Data was tabularized to be ready for statistical analysis, that was executed utilizing SPSS software, version 26. Means as well as standard deviations were determined and represented in MPa. Data were analyzed with two- and one-way ANOVA succeeded by Tukey’s post hoc test and student’s t-test (α = 0.05).

## Results

### Micro-tensile bond strength (µTBS) test

**Fig. 1:**
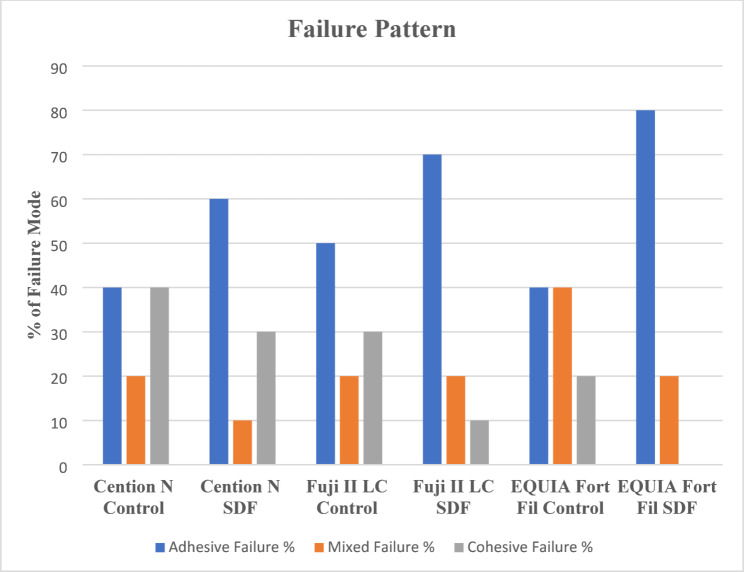
Different failure patterns % after µTBS showing high values of adhesive failures in SDF subgroups

Two-way ANOVA revealed no significant interaction between factors under the study (*P* > 0.05) (Table 2). Results of µTBS are summarized in Table 3. The type of restorative material significantly affected µTBS values within each group (one-way ANOVA, *P* ≤ 0.05). The highest result within control as well as SDF groups was for Cention N. Tukey’s post hoc test demonstrated that there was a statistically significant difference between Cention N and EQUIA Fort fil within both groups (*P* ≤ 0.05). However, no significant difference among Cention N and Fuji II LC was reported (*P* > 0.05). In the meantime, the least value within control and SDF groups was for EQUIA Fort Fil with no significant difference with Fuji II LC (*P* > 0.05). The results of t-test demonstrated a statistically significant reduction in µTBS after SDF application in all tested materials (*P* ≤ 0.05). The modes of failure are summarized in Fig. 1. Adhesive failure was the dominant failure mode among all groups, especially SDF subgroups.

**Table 2:** Two-way ANOVA showing the effect of restorative material type, SDF treatment, and their interaction on micro-tensile bond strength (µTBS)

Source of variation	DF	Sum of Squares	Mean Square	F-value	*P*-value
**Restorative Material**	2	1814.36	907.18	10.66	0.0001
**SDF**	1	8981.7	8981.71	105.50	< 0.0001
Restorative Material*SDF	2	22.69	11.34	0.13	0.8755
**Error**	54	4597.46	85.14		
**Total**	59	15416.22			

DF: Degree of Freedom

Statistically significant difference at *P* ≤ 0.05

**Table 3:** Mean micro-tensile bond strength values (µTBS, in MPa) and standard deviations (SD) for control and SDF-treated groups, including tukey’s and t-test comparisons

Group	Control		SDF		*P*-value
	Mean	SD	Mean	SD	
**Cention N**	45.84^a^	11.19	19.66^a^	12.01	0.0001
**EQUIA Fort Fil**	31.23^b^	9.11	7.34^b^	3.83	0.0001
**Fuji II LC**	38.00^ab^	9.75	14.66^ab^	6.97	0.0001
**P-value**	0.0115		0.0096		

SD: Standard Deviation

a-b = Means with the identical letters in each column are not significantly different at *P* ≤ 0.05 with Tukey test

### Evaluation of dentine/restorative materials interface

**Fig. 2:**
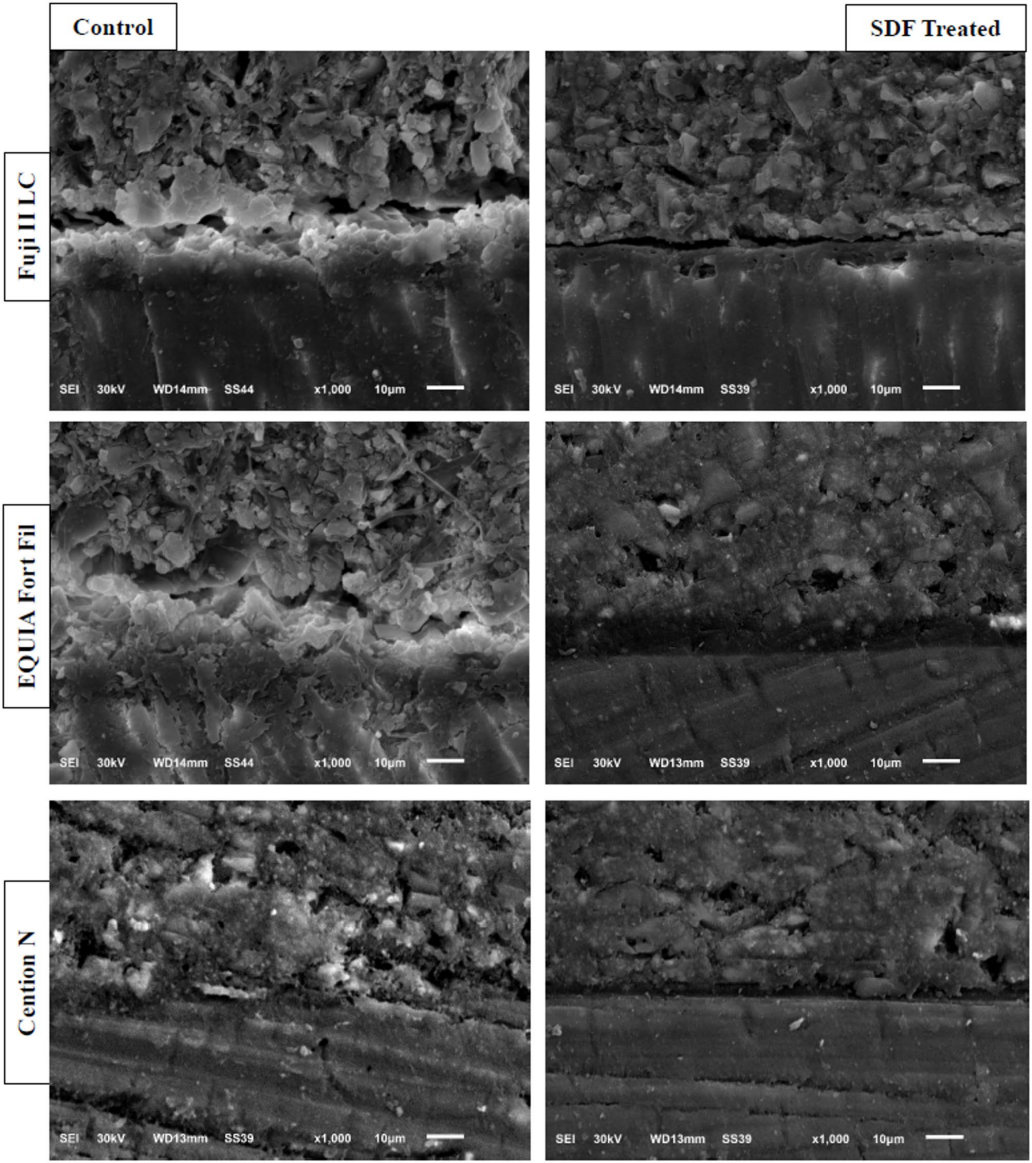
SEM Micrographs of the dentine/restorative materials interface of Control and SDF treated groups

**Fig. 3:**
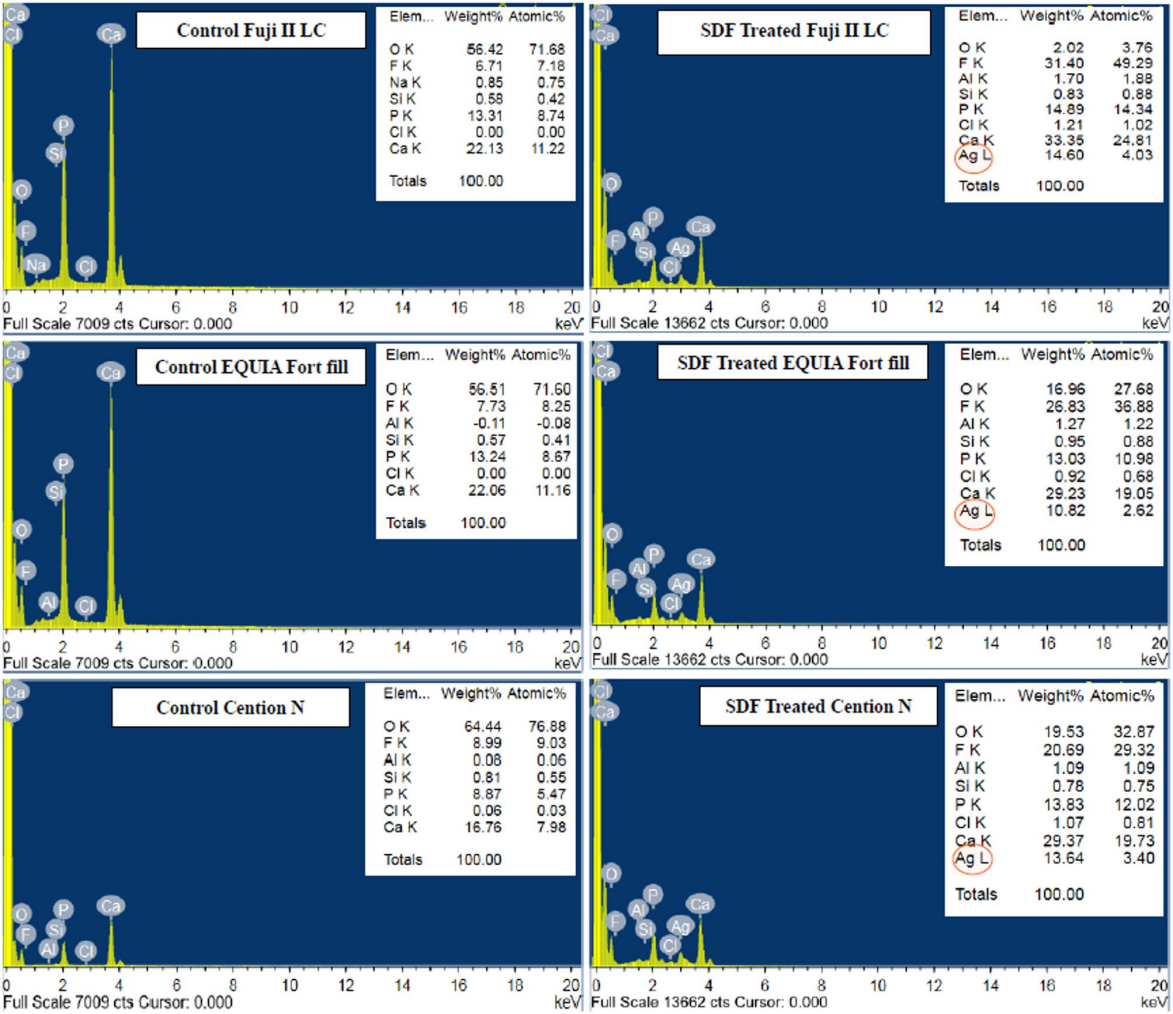
EDX analysis of dentinal tubules close to the interface. The increased intensities of Ag and Ca peaks confirm the presence of silver particles following SDF treatment

Figure 2. displays the SEM micrographs obtained using backscattered electron detection to each group at ×1,000 magnification. Figure 3. presents the results of EDX analysis conducted on dentinal tubules near the interface. For control subgroups, in which no SDF was applied, the dentine/restorative materials interface appears continuous, featuring a well-formed interdiffusion zone across the interface. EDX analysis of the dentinal tubules adjacent to dentine/restorations interfaces confirmed no detection of silver precipitates. For SDF subgroups, the dentine/restorative materials interface exhibits greater discontinuity with microgabs formation across the interface besides exhibiting dense surface precipitates with no detectable interdiffusion zone. EDX analysis of the dentinal tubules adjacent to dentine/restorations interfaces indicated the presence of silver compounds. Furthermore, SDF treatment resulted in a notable increase in the calcium and fluoride peaks.

## Discussion

In this contemporary study, SDF application directly in advance of restoration significantly reduced the µTBS of the three tested restorative materials to artificially demineralized dentine after thermocycling. Additionally, significant differences in bond strength were observed among the restorative materials, irrespective of SDF treatment. Accordingly, the null hypothesis was rejected. This outcome has critical clinical implications, highlighting the necessity for optimizing bonding protocols when SDF is applied prior to restoration to ensure durable adhesion and restoration longevity.

Achieving an optimal peripheral seal is crucial for the durability of restorations. However, the impact of SDF pretreatment on the bond strength of restorative materials to dentine remains a topic of debate. Thus, this study inspected the impact of 38% SDF treatment on microtensile bond strength of three bioactive materials: GHR, RMGI and Alkasite. There are diverse commercially presented SDF concentrations utilized in dentistry including 12%, 30% and 38%. 38% SDF was selected for the contemporary study consistent with Fung et al. [47] who settled that 38% SDF was statistically noteworthy in caries halting and prevention compared with 12% SDF [47]. The bond strength durability was evaluated using artificially demineralized dentine produced through a pH-cycling model. This demineralization-remineralization approach provides a controlled and reproducible means of simulating clinically relevant leathery or firm dentine found in carious lesions. Previous studies have demonstrated that it produces dentine with physical and chemical properties (e.g., hardness and permeability) comparable to those observed in natural leathery or firm dentine found in carious lesions [48, 49]. This model was chosen to standardize substrate conditions and reduce variability, allowing for more reliable assessment of bonding performance [49, 50].

Numerous restorative materials serve as an aid in remineralizing demineralized teeth procedure. Fluoride release with other elements could be decisive in this remineralization process. Glass ionomer restorations are regularly considered the primary materials in this regard. Additionally, Cention N was used in this study due to its ion-releasing glass fillers (alkaline filler) besides having compressive strength and diametral tensile strength (DTS) surpassing than that of glass ionomer [51]. To the best of the authors’ knowledge, the current evidence remains limited regarding the bond strength of alkasite to SDF treated dentine besides the impact of aging on bond strength need to be more explorated.

Group I (control group) in this study showed higher significant µTBS than group II (SDF treated). Within Group I, the highest bond strength was reported in alkasite group followed by RMGI then GHV. The higher bond strength of alkasite could be clarified by its unique monomer composition and optimized resin formulation. Alkasite materials such as Cention N do not contain conventional monomers like Bis-GMA, HEMA, or TEGDMA; instead, they primarily incorporate urethane dimethacrylate (UDMA) as the core monomer. This is complemented by other components such as decandiol dimethacrylate (DCP), aromatic and aliphatic UDMA, and polyethylene glycol dimethacrylate (PEG–400 DMA) [52]. This combination contributes to a high polymer network density, enhanced mechanical properties, and improved long-term stability during polymerization. PEG–400 DMA, in particular, is a hydrophilic, low-viscosity monomer that increases the material’s fluidity and supports effective wetting and adaptation to enamel and dentin surfaces, including interaction with the smear layer [52]. These characteristics collectively enhance micromechanical interlocking and interfacial adhesion, which may explain the superior microtensile bond strength of alkasite materials when compared to resin-modified glass ionomer cements [53].

RMGICs demonstrated better bonding performance compared to conventional glass ionomer cements (GICs), primarily due to their modified composition and dual-setting mechanism. Comprising both traditional glass ionomer and resin components, RMGICs benefit from the simultaneous action of an acid-base reaction and light-activated resin polymerization. The acid-base component facilitates chemical bonding through ionic interaction with the tooth structure, while the resin polymerization promotes micromechanical interlocking by allowing resin infiltration into the dentin surface [54]. This dual-curing mechanism enhances the material’s adaptation to dental substrates, improves early mechanical strength, and reduces moisture sensitivity during the initial setting phase [55, 56]. Additionally, the inclusion of hydrophilic resin components such as hydroxyethyl methacrylate (HEMA) enhances mechanical properties and promotes micromechanical interlocking and interfacial adhesion to dentin and enamel, in addition to the ionic bonding characteristic of conventional GICs [55, 57].

The lower µTBS of SDF group compared to control group could be attributed to the SDF basicity (pH = 10), that might obstruct polyacrylic acid from eradicating dentine surface smear layer preceding to RMGI or GHR insertion [38]. Moreover, SDF basicity could impact the acid-base reaction of GIC through hindering calcium and aluminum ions release at the dentine-GIC interface with subsequent reduction in bond strength [38]. Moreover, the alkaline nature of the SDF solution creates an ideal environment for covalent bonds to form between phosphate groups and collagen fibrils, facilitating the incorporation of phosphate into the collagen. The phosphorylation process creates a negatively charged dentine surface that attracts calcium ions via electrostatic forces, facilitating the growth of hydroxyapatite crystallites. The enhanced availability of binding sites for phosphate and calcium ions initiates nucleation, leading to the growth of HA crystals and an increase in mineral content on the dentine surface, which likely explains the high intensity of the calcium peaks detected during EDX-SEM analysis [46].

The existence of dense precipitates on the SDF-treated dentine surface is another possible theory [4, 38]. SDF could interact with calcium and phosphate ions to form calcium fluoride or fluorohydroxyapatite crystals. These dense less soluble precipitates could inhibit tag formation, limiting micromechanical interlocking [38, 58]. Besides, the remaining silver layer from SDF might change the surface energy and the dentine surface wettability, which in turn can negatively affect adhesion of restorative materials to the dentine underneath. Moreover, this layer most likely hinders intimate contact between restoration and dentine [43]. Consequently, the carboxylate groups in the RMGI or GHR, as well as the resin in Alkasite have fewer probabilities to interact with calcium ions from the hydroxyapatite [43].

These results are in line with a number of studies which showed a reduction in bond strength after SDF application [10, 38, 39, 59]. In the meantime, other studies concluded that the bond strength is not affected or even increased after SDF application [32–35, 60, 61]. This could be due to different application protocols or different sample sizes in addition to the absence of aging in these studies. Additionally, unlike previous studies where SDF-treated dentine surface was polished using silicon carbide papers [35, 45], the SDF treated dentine surface in this contemporary study was only rinsed using water spray with no polishing procedure preceding restoration application. This approach was used to imitate the ART clinical scenario, where the SDF-treated dentine surface is not touched prior to cavity restoration.

The clinical implication of the study findings is important. It is revealed that if dentine surfaces have been treated using SDF solution, the consequent inserted restoration might debond quickly resulting in premature restoration failure. Therefore, it is suggested to gently grind the SDF-treated dentine surfaces using a diamond bur to eliminate the superficial precipitated layer. Furthermore, restorative materials ought to be bonded to sound dentin at cavity margins to optimize overall bond strength.

Some limitations of this study should be considered. This contemporary work was accomplished under precise laboratory conditions, where dentine was artificially demineralized using pH-cycling model. While this method provides controlled and reproducible conditions, it does not fully replicate the complex biological environment of natural carious dentine, particularly the presence of bacterial invasion, collagen matrix degradation, and the incomplete chemical simulation of the oral cavity [50, 62]. Moreover, dentine demineralized using this model tends to exhibit more uniform mineral loss and shallower lesion depth, which may not accurately represent the heterogeneous structural and chemical characteristics of clinically encountered carious lesions [63]. Consequently, the two distinct substrates may provide different circumstances that will most probably result in varying adhesive qualities. An additional limitation of this study is the high variability in µTBS values among SDF-treated groups, likely due to the heterogeneous nature of demineralized dentine and the non-uniform interaction of SDF with the substrate. Besides, bond strength testing is inherently influenced by multiple factors, including complex dentine structure, tooth age, dentine depth, tubule density and moisture control. Future in vivo studies and long-term evaluations are recommended to validate these findings and further optimize restorative protocols following SDF application.

## Conclusions

Based on the limitations of this laboratory study, the application of SDF (38%) directly preceding restoration with RMGI, GHR or Alkasite, even with water rinsing step after SDF application, resulted in a significant deterioration in bond strength to artificially demineralized dentine. These findings suggest that the interaction between SDF-treated substrates and various restorative materials may compromise adhesion, highlighting the need for further investigation into optimized clinical protocols.

## Data Availability

The datasets generated and/or analyzed during the current study are available from the first author Mostafa A. Abdelshafi upon request.

## Abbreviations

SDF: Silver Diamine Fluoride
ART: Atraumatic Restorative Technique
CAD: Caries Affected Dentine
ACAD: Artificially Induced Caries Affected Dentine
DD: Demineralized Dentine
GIC: Glass ionomer Cement
PAA: Polyacrylic Acid
RMGIC: Resin Modified Glass Ionomer Cement
HEMA: 2-hydroxyethylmethacrylate
GH: Glass Hybrid
GHR: Glass Hybrid Restorative
CN: Cention N
UDMA: Urethane-Dimethacrylate
DCP: Tricyclodecan-Dimethanol- Dimethacrylate
PEG-400 DMA: Polyethylene-Glycol 400-Dimethacrylate
µTBS: Microtensile Bond Strength
EDX: Energy-Dispersive X- ray
SEM: Scanning Electron Microscope
DTS: Diametral Tensile Strength
FDA: Food & Drug Administration
